# Mito-TEMPO improves development competence by reducing superoxide in preimplantation porcine embryos

**DOI:** 10.1038/s41598-018-28497-5

**Published:** 2018-07-04

**Authors:** Seul-Gi Yang, Hyo-Jin Park, Jin-Woo Kim, Jae-Min Jung, Min-Ji Kim, Ho-Guen Jegal, In-Su Kim, Man-Jong Kang, Gabbine Wee, Hee-Young Yang, Yun-Han Lee, Ji-Hae Seo, Sun-Uk Kim, Deog-Bon Koo

**Affiliations:** 1 Department of Biotechnology, College of Engineering, Daegu University, 201 Daegudae-ro, Jillyang, Gyeongsan, Gyeongbuk, 38453 Republic of Korea; 2 Department of Animal Science, College of Agriculture and Life Sciences, Chonnam National University, Gwangju, 61186 Republic of Korea; 3 Laboratory Animal Center, Daegu-Gyeongbuk Medical Innovation Foundation (DGMIF), 80 Cheombok-ro, Dong-gu, Daegu, 41061 Republic of Korea; 4 Department of Molecular Medicine, Keimyung University School of Medicine, Daegu, 42601 Republic of Korea; 5 Department of Biochemistry, Keimyung University School of Medicine, Daegu, 42601 Republic of Korea; 6 National Primate Research Center & Futuristic Animal Resource and Research Center, Korea Research Institute of Bioscience and Biotechnology, Ochang, Chungbuk 28116 Republic of Korea

## Abstract

Mito-TEMPO is a well-known mitochondria-specific superoxide scavenger. However, the effect of Mito-TEMPO on porcine embryo development, to our knowledge, has not been studied yet. In the present study, porcine embryos were classified into two groups (G1 and G2) based on the cytoplasm lipid contents at the zygote stage. The development of blastocysts derived from G2 zygotes was reduced (G2:16.2 ± 7.9% vs G1: 26.5 ± 5.9%; 1.6-fold, p < 0.05) compared to those from G1 zygotes. In G2 embryos, the proportion of TUNEL-positive cells was also higher than that of G1 embryos. Superoxide in G2 embryos was significantly increased compared to that in G1 embryos. Mitochondrial membrane potential and ATP production were lower in G2 embryos than in G1 embryos. Phosphorylation of Drp1 at Ser 616 increased in G1 embryos during the cleavage stages compared to that in the zygote but was not significantly different in G2 embryos. Then, the effects of Mito-TEMPO were investigated in G2 embryos. Blastocyst formation rate (G2: 19.1 ± 5.1% vs G2 + Mito-TEMPO: 28.8 ± 4.0%; 1.5-fold, p < 0.05) and mitochondrial aggregation were recovered after superoxide reduction by Mito-TEMPO treatment. Thus, we showed that Mito-TEMPO improves blastocyst development by superoxide reduction in porcine embryos *in vitro*.

## Introduction

Mitochondria are well-known organelles for adenosine triphosphate (ATP) production, which is important for controlling cell growth, signaling, dynamic response, and apoptosis in most mammalian cells. In porcine oocytes and/or embryos, a high level of ATP production in the cytoplasm is necessary for oocyte maturation, fertilization, and early embryo development *in vivo* and *in vitro*^1^. Particularly, after *in vitro* fertilization (IVF), zygote stage porcine embryos contain high levels of lipids consisting of triglycerides for energy storage^2^. Many studies have shown that ATP production-related lipid metabolism is important for early embryo development^3,4^.

During ATP production, reactive oxygen species (ROS) such as hydrogen peroxide, superoxide, and hydroxyl radicals are generated by oxidative phosphorylation in mitochondria^5^. This production of ROS is linked to oocyte maturation, fertilization, and embryo development in pigs^6^. Many studies have shown that ROS accumulation reduces embryonic developmental competence and blastocyst quality in pigs^7^, cattle^8^ and mice^9^. In addition, an imbalance between free radical formation and removal can lead to oxidative stress, which can induce DNA damage and increase the expression of pro-apoptotic genes leading to cell death during oocyte maturation and early embryonic development^10,11^.

Moreover, severe oxidative stress resulting from increasing ROS is known to induce mitochondrial fission that elicits mitochondria’s dynamic response^12^, aggregation^13^ and dysfunctions^14^. Mitochondrial fission, one of the mitochondrial dynamic responses, is regulated by the fission protein, dynamin-related protein 1 (Drp1). Increasing mitochondrial fission by the accumulation of ROS reduced the ATP production and mitochondrial membrane potential (MMP)^15^. This, in turn, induced mitochondrial-derived apoptosis as a result of dysfunction of MMP under conditions of ROS accumulation^16^.

Most of all, superoxide derived from mitochondrial ROS can act as signaling molecules to activate pro-growth responses in cancer cells^17^. It was recently proven that triphenylphosphonium chloride (Mito-TEMPO) is a superoxide scavenger and a physiochemical compound mimicking superoxide dismutase from mitochondria. It can easily pass through the lipid bilayers and accumulate in the mitochondria^18^. Mito-TEMPO has been implicated in the functions of mitochondria because it has the role of eliminating mitochondrial ROS. For instance, Mito-TEMPO prevents oxalate-induced injury by inhibiting mitochondrial dysfunctions and modulating oxidative stress in NRK-52E cells^19^ and inhibition of mitochondrial ROS with Mito-TEMPO reduced diabetic cardiomyopathy^18^. However, the effects of Mito-TEMPO on porcine embryo development has not been investigated yet.

In order to understand the effects of Mito-TEMPO on porcine embryo development, we divided fertilized zygotes into two groups (high cytoplasm components: grade 1, G1 and low cytoplasm components: grade 2, G2) based on the cytoplasm components of the zygotes. Further, we investigated the changes in expression of the mitochondria-derived superoxide on porcine embryos preimplantation development in G1 and G2 groups using the mitochondrial superoxide-specific staining indicator, Mito-SOX. Subsequently, we measured the maintenance of mitochondrial functions and regulation of dynamic response in the early development of porcine embryos. Subsequently, effects of Mito-TEMPO as a superoxide scavenger were assessed by examining the embryo development rate as well as the distribution of mitochondria. Here, we show how superoxide reduction is linked to better embryo development in porcine using Mito-TEMPO, which changes the mitochondrial ROS and distribution.

## Results

### Developmental competence until blastocyst stage for G1 and G2 porcine zygotes

**Figure 1:**
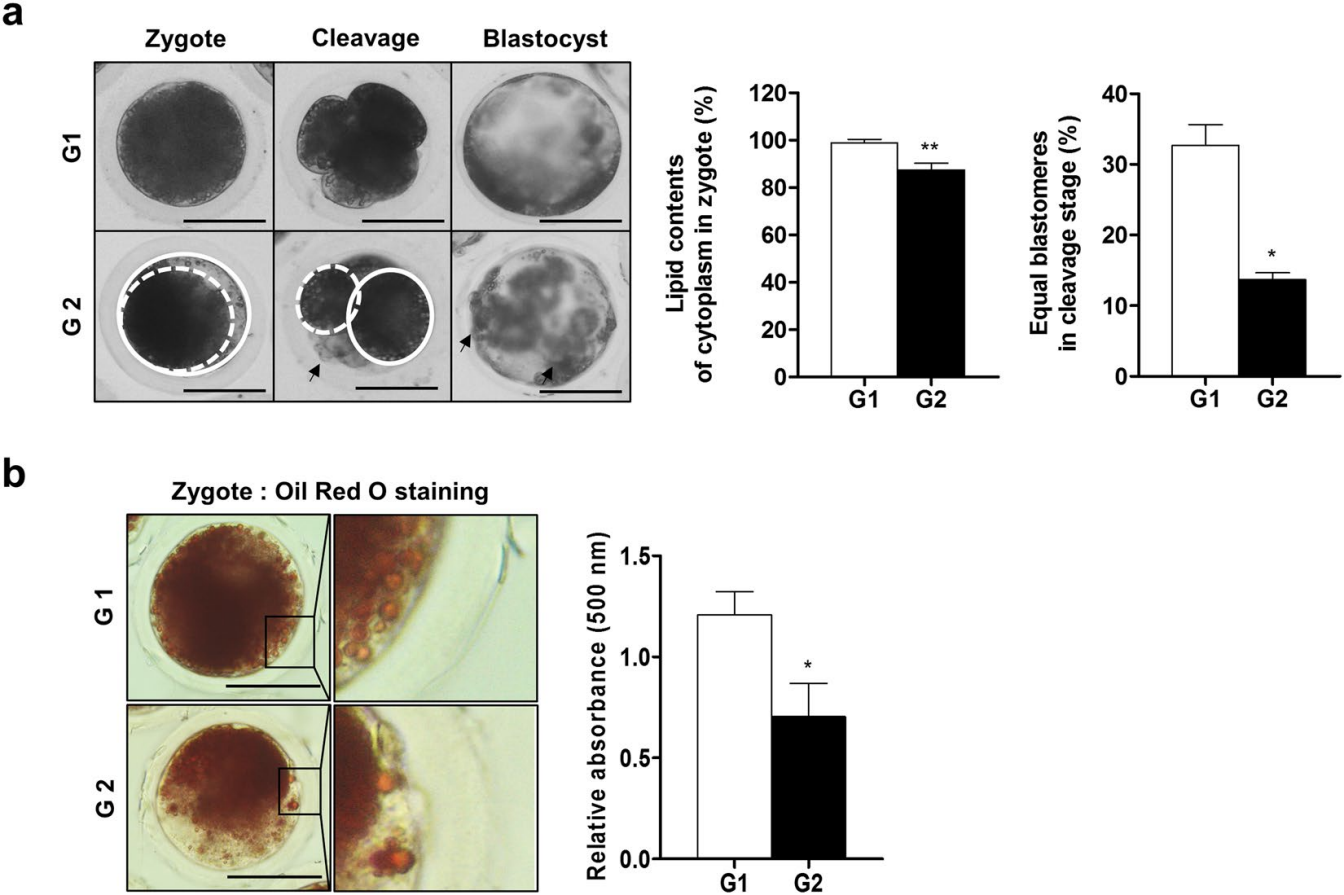
Morphology of embryos according to the cytoplasm area. (**a**) Morphology at each embryo stage was visualized using an optical microscope (left panel). White lines represent the area of embryo cytoplasm and arrows indicate fragments. Quantitative analysis of cytoplasm and equal-sized blastomeres of cleavage stage in G1 and G2 embryos (right panel). (**b**) Stained lipid droplets of zygote were visualized using an optical microscope (left panel). Extracted Oil Red O reagents were measured at 500 nm wavelength using a microplate reader (right panel). Data in the bar graph represent the means ± SEM from three independent experiments. To analyze these data, a *t*-test was used. Differences were considered significant at *p < 0.05, ** < 0.01. Scale bar = 100 μm.

As shown in Fig. 1a, we divided the zygotes into two groups (grade 1, G1: high cytoplasm components; grade 2, G2: low cytoplasm components) according to the cytoplasm and morphology of the zygotes at stages described by Van Soom *et al*., Ajduk, and Zernicka-Goetz^20,21^. To confirm the difference in the cytoplasm components of the zygotes, we measured lipids as triglycerides using the Oil Red O stain. As expected, the values of isopropanol absorbance spectrum measurements in extracted lipid derived from zygote cytoplasm significantly increased (p < 0.05) in the G1 zygote group compared to G2 zygotes (Fig. 1b). In addition, lipid droplet size in G1 zygote was smaller than G2 zygote. In the cleaved stage, we also observed the increase of large lipid droplets in G2 group embryos than that of G1 group embryos (data not shown). Moreover, the development rate of blastocysts from G2 zygotes was significantly reduced (1.8-fold, p < 0.05, G2: 16.2 ± 7.9% vs G1: 26.5 ± 5.9%) compared to G1 zygotes (Table 1). Therefore, embryos of G1 and G2 groups were classified based on the cytoplasm lipid contents at the zygote stage, and embryos developed from two groups showed the different developmental potential until blastocyst stage. Based on these results, embryos of G1 and G2 groups could be used for subsequent experiments on porcine embryo development.

**Table 1:** Development rate according to lipid of cytoplasm at zygote.

Groups	No. of embryos cultured	% of embryos cleaved (n)	% of blastocysts (n)
Grade 1	290	78.4 ± 14.1 (227)	26.5 ± 5.9 (78)^a^
Grade 2	268	67.5 ± 15.3 (192)	16.2 ± 7.9 (44)^b^

Data are expressed as means ± SD of three independent experiments. Different superscript letters a and b denote significant differences (p < 0.05).

### Detection of intracellular ROS, superoxide, and cellular apoptosis during the preimplantation development of G1 and G2 embryos

**Figure 2:**
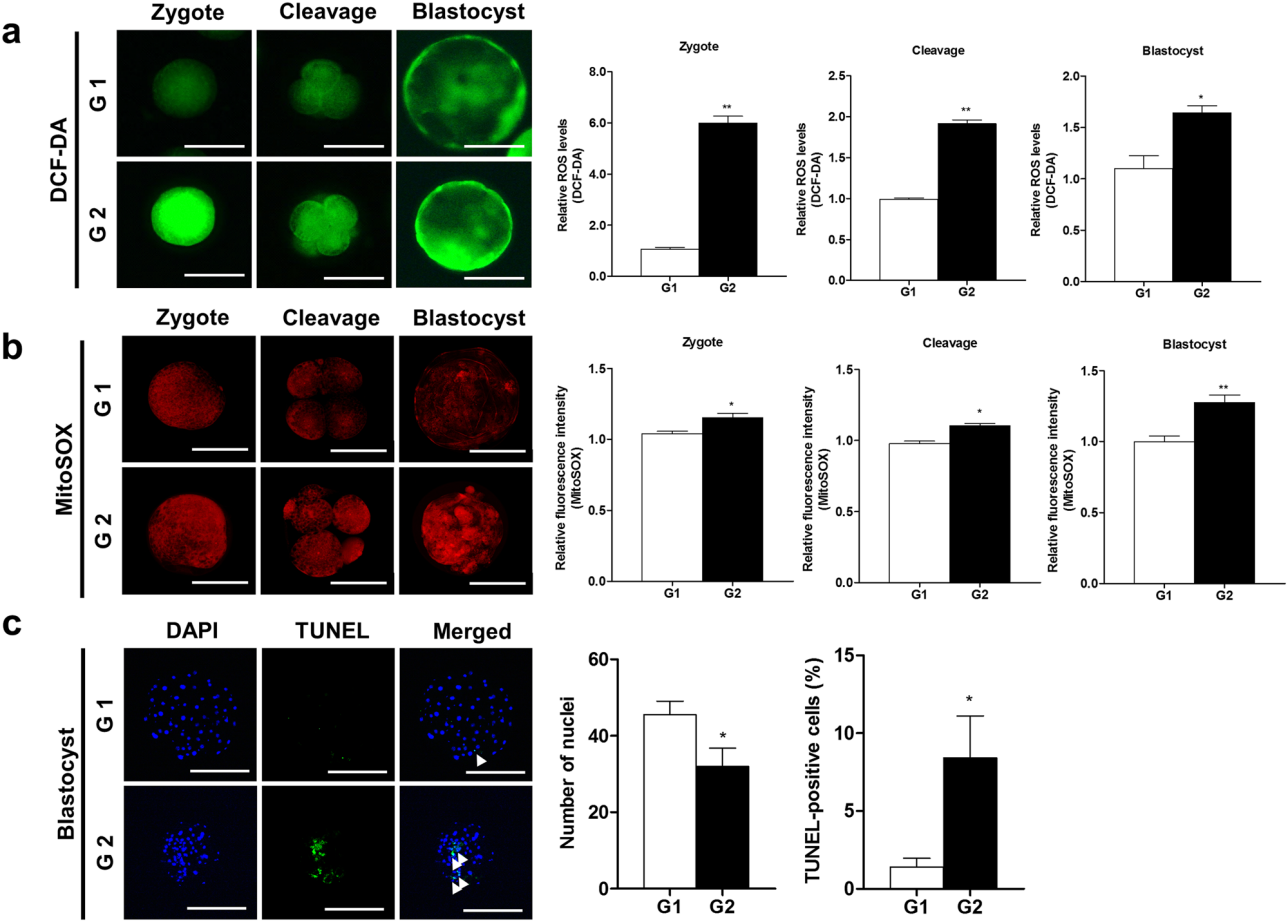
Changes in intracellular ROS, superoxide, and apoptosis according to the grade in preimplantation embryos. (**a**) Expression of intracellular ROS detected by DCF-DA (green) and analyzed using epifluorescence microscope. (**b**) MitoSOX (red) as superoxide indicator detected using a confocal microscope. (**C**) DAPI (blue) and TUNEL-positive cells (green) of blastocysts shown using a fluorescence microscope. The arrows indicate apoptotic cells in nuclei. Data in the bar graph represent the means ± SEM from three independent experiments. To analyze these data, a *t*-test was used. Differences were considered significant at *p < 0.05 and ** < 0.01. Scale bar = 100 μm.

We measured intracellular ROS and superoxide levels using dichlorofluorescin diacetate (DCF-DA) (green fluorescence) and MitoSOX (red fluorescence) as mitochondrial superoxide indicators in G1 and G2 embryos, respectively. Intracellular ROS levels drastically increased (zygote: p < 0.01 and cleavage/blastocyst: p < 0.05) until the blastocyst stage in the embryos of G2 (Fig. 2a). Simultaneously, red fluorescence of superoxide expression was also significantly increased (p < 0.05) in the G2 embryos during preimplantation stages (Fig. 2b). In addition, we detected apoptotic cells using the 4’,6-diamidino-2-phenylindole (DAPI) and terminal deoxynucleotidyl transferase-mediated dUDP nick-end labelling (TUNEL) assay in blastocysts from the G1 and G2 groups (Fig. 2c). The number of nuclei in blastocysts was reduced (p < 0.05), while the percentage of TUNEL-positive cells increased (p < 0.05) in developed blastocysts from the G2 compared to the G1 group. These results showed that the mitochondrial ROS level of superoxide increased in the porcine embryo developmental stage.

### Changes in mitochondrial function and distribution in embryos of G1 and G2 groups

**Figure 3:**
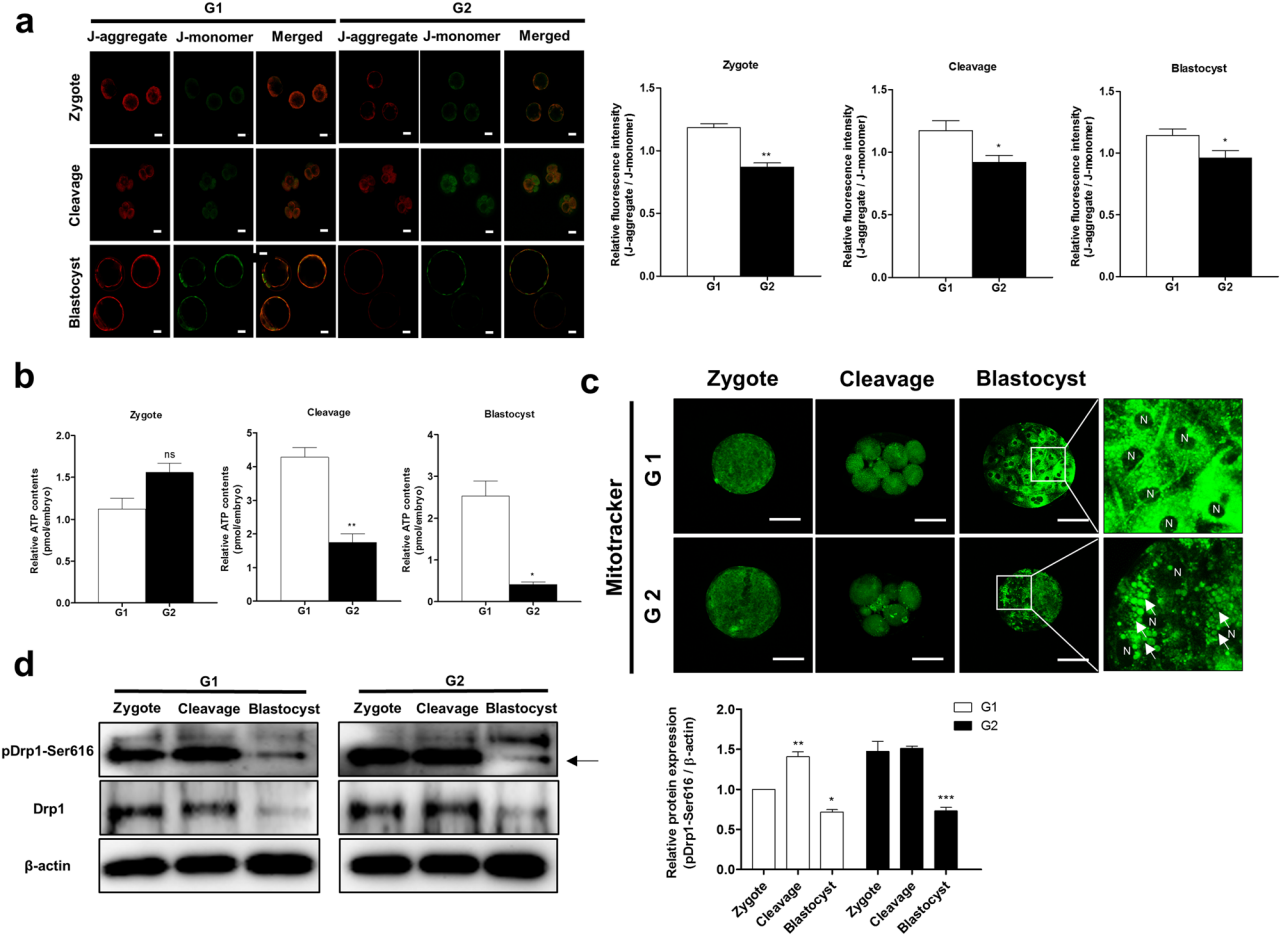
Changes in mitochondrial function and dynamics in the G1 and G2 groups. (**a**) Expression of J-aggregate (red) and J-monomer (green) were determined by JC-1 using a confocal microscope. (**b**) ATP contents were measured by an ATP determination kit using a microplate reader. (**c)** Mitotracker (green) as the mitochondria-selective probe was measured under image system. N indicates the nucleus and arrows represent mitochondrial aggregation form. (**d**) Cropped blots are used in this figure, and the gels have been run under the same experimental conditions. Full-length blots are included for key data in the supplementary information. Western blot showing expression of mitochondrial fission proteins (Drp1 and phosphorylated Drp1-Ser616). Reactive protein levels were normalized to β-actin. Data in the bar graph represent means ± SEM from three independent experiments. To analyze these data, Dunnett’s multiple comparison test and *t*-test were used. Differences were considered significant at *p < 0.05 and ** < 0.01. Scale bar = 50 μm.

To investigate the changes in the mitochondrial functions of G1 and G2 porcine embryos, we measured the MMP and ATP production using JC-1 and an ATP determination kit, respectively. The red fluorescence of MMP in G2 embryos significantly decreased during the zygote (p < 0.01), cleavage (p < 0.05), and blastocyst (p < 0.05) stages (Fig. 3a). ATP production in G2 embryos was significantly reduced compared to G1 embryos during the cleavage (p < 0.01) and blastocyst (p < 0.05) stages (Fig. 3b). In addition, we observed the mitochondrial aggregation and distribution using Mitotracker staining (green fluorescence expression) during different developmental stages of porcine G1 and G2 embryos. As shown in Fig. 3c, G1 blastocysts showed an even distribution of mitochondria, contrary to the G2 blastocysts. Furthermore, in the case of G2 blastocysts, most mitochondria were aggregated. To investigate the expression pattern of mitochondrial fission proteins (Drp1 and its phosphorylated form: pDrp1-Ser616) in G1 and G2 embryos, we also performed Western blot analysis (Fig. 3d). This showed that pDrp1-Ser616 protein expression levels in G2 embryos declined steadily from the zygotic stage until the blastocyst stage (p < 0.001), whereas the expression levels of the same protein significantly increased (p < 0.01) in the G1 cleaved embryos compared to other development stages. These results demonstrated that the mitochondrial functions, distribution pattern, and fission protein levels are associated with porcine embryonic development.

### Effects of Mito-TEMPO on preimplantation porcine embryo development

**Figure 4:**
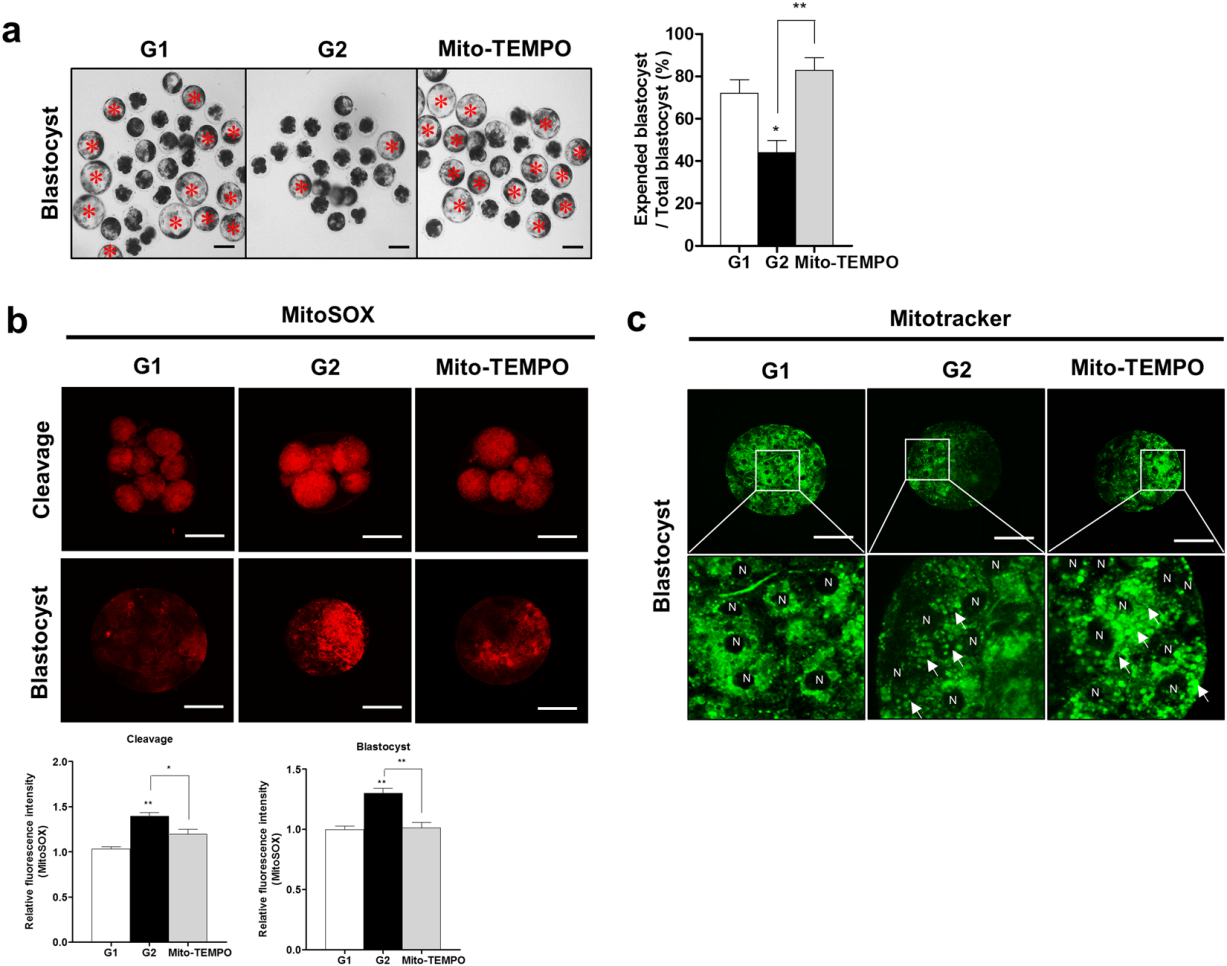
Effects of Mito-TEMPO on early embryo development in porcine. (**a**) Red stars indicate the number of expanded blastocysts. Morphologies of blastocyst stage in G1, G2 and G2 with Mito-TEMPO treatment. Detection of (**b**) superoxide (red fluorescence) and (**c**) mitochondrial distribution (green fluorescence) in G2 zygotes treated with Mito-TEMPO during embryo development. The arrows show aggregation of mitochondria and N represents the nucleus. Data in the bar graph represent the means ± SEM from three independent experiments. To analyze these data, Tukey’s multiple comparison test was used. Differences were considered significant at *p < 0.05 and ** < 0.01. Scale bar = 50 μm.

First of all, we confirmed the meiotic maturation by using orcein staining in matured porcine oocytes according to various concentrations (0.1, 0.5 and 1.0 µM) of Mito-TEMPO (Supplementary Table S1). As a result, the meiotic maturation rate of porcine oocytes was significantly increased (p < 0.05) in 0.1 µM Mito-TEMPO treated group compared with other groups. Based on this result, we determined the optimal concentration of 0.1 µM Mito-TEMPO for porcine embryos development in present study. We investigated the effects of Mito-TEMPO on porcine embryo development using the G1 and G2 zygotes. As shown in Fig. 4a, expanded blastocyst production was significantly increased (p < 0.01) in Mito-TEMPO-treated G2 embryos compared with the G2 group. Developmental competence in G2 embryos was recovered (p < 0.05, G2 + Mito-TEMPO: 28.8 ± 4.0% vs G2: 19.1 ± 5.1%) by 0.1 μM Mito-TEMPO treatment (Table 2). This was accompanied by a significant reduction in the red fluorescence expression of superoxide in developed blastocysts from G2 embryos treated with 0.1 μM Mito-TEMPO (cleavage: p < 0.05 and blastocyst: p < 0.01) compared to untreated G2 embryos (Fig. 4b). As shown in Fig. 4c, mitochondrial aggregation in Mito–TEMPO-treated blastocysts were also reduced compared with blastocysts from untreated G2 embryos. These results showed positive effects of Mito-TEMPO treatment in improving the blastocyst developmental ability through reducing mitochondrial superoxide in porcine embryos.

**Table 2:** Development rate by Mito-TEMPO treatment.

Groups	No. of embryos cultured	% of embryos cleaved (n)	% of Blastocysts (n)
Grade 1	330	75.2 ± 6.9 (250)	27.8 ± 4.0 (70)^a^
Grade 2	304	67.1 ± 19.4 (213)	19.1 ± 5.1 (56)^b^
Grade 2 + Mito-TEMPO	306	74.7 ± 15.2 (231)	28.8 ± 4.0 (89)^a^

Data are expressed as means ± SD of three independent experiments. Different superscript letters a and b denote significant differences (p < 0.05).

## Discussion

In the present study, we investigated if Mito-TEMPO, a mitochondrial superoxide scavenger, improves the quality and developmental competence of blastocysts by regulating mitochondrial functions in the porcine embryo. To do this, we divided the early embryos into two groups (G1 and G2) by measuring the lipid content using Oil Red O staining. As expected, blastocyst development rate and quality varied according to the lipid contents at different zygote stages. Based on these results, we investigated mitochondrial functions, distribution and superoxide production from mitochondria in G1 and G2 porcine embryos. We found that the blastocysts development rate, total cell number, MMP, and ATP significantly decreased in the G2 group compared to G1 group. On the other hand, intracellular ROS, superoxide, apoptotic cells, and mitochondrial aggregation increased in the G2 group. In addition, we confirmed the effects of superoxide reduction by Mito-TEMPO treatment on early embryo development in porcine. Blastocyst development of Mito-TEMPO-treated G2 group significantly increased, which was accompanied by decreasing superoxide and mitochondrial aggregation in cleaved embryos and blastocyst. These results confirmed that Mito-TEMPO had superoxide reduction effect, which is important for the regulation of mitochondrial functions and improvement of the quality and development potential of the porcine embryo.

Lipids in the cytoplasm of oocytes and embryos are key energy sources for bovine^22^ and porcine embryo metabolism^2^ during *in vitro* or *in vivo* development. The triglyceride constituting the lipid droplet is composed of one glycerol and three fatty acids. A recent study showed that one molecule of fatty acid can produce more energy compared to glucose oxidation in mammalian oocytes^4^. Thus, lipids are important in generating energy for supporting oocyte maturation and embryo development. Oil Red O stains neutral fats containing triglycerides in lipid droplets^23^. Small size lipid droplets is known to supporting oocyte maturation^4^. To confirm the cytoplasm lipid contents of porcine zygote, we used the Oil Red O staining method for containing triglycerides quantity in lipid droplets. As shown in Fig. 1b, extracted lipids and small size lipid droplets in zygote cytoplasm were increased in the G1 zygote group compared to G2 zygote. We also confirmed that the reduction of lipid droplets formation showed in Mito-TEMPO treated G2 embryos compared with G2 groups during cleaved stage (data not shown). It is well known that mitochondrial dysfunctions and ROS-derived oxidative stress induce the increasing of lipid droplets formation in glia, as well as lipid droplets accumulation on cytoplasm also occurs mitochondria defects in mice^24^. Additionally, rapid accumulation or large size of cytoplasmic lipid droplets is induced mitochondrial dysfunctions under cellular apoptosis^25^. Although the detailed mechanism of between lipid droplets and mitochondria functions according to the Mito-TEMPO treatment was not investigated in present study, the mitochondria functions and lipid droplets formation may be involved in superoxide reduction by Mito-TEMPO in porcine embryos. Moreover, the development rate of blastocysts decreased in G2 blastocysts compared to G1 (Table 1). Taken together, our results suggest that the contents of lipids in zygotes affect embryonic development.

ROS is produced in mitochondria during early porcine embryo development. Particularly, ROS functions as an intracellular signaling molecule in embryo metabolism^11^. However, excess oxidative stress can lead to mitochondrial dysfunctions, DNA damage, delay of embryo development, inhibition of oocyte maturation, and induction of apoptosis of embryos^26^. As shown in Fig. 2, we confirmed that intracellular and mitochondrial ROS and apoptosis increased in the G2 group compared to the G1 group. Moreover, a recent study demonstrated that the superoxide regulation in mitochondria is related to mitochondrial dysfunctions and cellular apoptosis mechanisms in cancer cells^27^. These results suggest that increase in oxidative stress is associated with a decrease in porcine embryo development.

High levels of ROS can lead to inhibition of mitochondrial metabolism and dysfunctions^28^. The mitochondrial health can be confirmed by MMP. Embryos have deficiencies of mitochondrial MMP by impair of oxidative phosphorylation and electron transport that could injured embryonic developmental competence^29^. Our results showed that MMP and ATP production levels were reduced in G2 embryos compared to G1 embryos (Fig. 3a,b). These results support the notion that mitochondrial dysfunctions occur because of excess oxidative stress in porcine embryos during early embryo development. Mitochondrial aggregation dramatically increased in G2 blastocysts compared to G1 blastocysts (Fig. 3c). According to our results, mitochondrial aggregation increased in G2 embryos. A previous study is known to mitochondrial aggregation precedes the release of cytochrome c from the mitochondria during apoptosis^30^. G2 embryos were showed decreasing embryonic development and increasing TUNEL-positive cells in blastocysts (Table 1 and Fig. 2c). We investigated the protein expression levels of mitochondrial fission factors in embryos derived from G1 and G2 zygotes. Western blotting results (Fig. 3d) showed that expression of mitochondrial fission protein pDrp1-Ser616 in the G2 group remained constant from the zygotic stage until the cleavage stage while it significantly increased during the cleavage stage for G1 group embryos. A previous study has shown that the expression pattern of pDrp1-Ser616 in zygotes with appropriate mitochondrial fission status was increased at the cleavage stage embryos^31^. These results suggest that mitochondrial functions and dynamics influence the competence of embryo development.

Mito-TEMPO, a mitochondrial superoxide scavenger, blocks mitochondrial ROS and intracellular ROS generation in hypertension^32^ and melanoma cells^33^. In addition, Mito-TEMPO preserves the mitochondrial integrity and attenuates stress-induced necrosis and apoptosis^34,35^. Therefore, we studied the blastocysts development rate and mitochondrial functions in Mito-TEMPO-treated G2 zygotes. As shown in Table 2, superoxide reduction by Mito-TEMPO improved blastocysts development rate in G2 embryos. Moreover, numbers of expanded blastocysts in G2 embryos treated with Mito-TEMPO also increased compared to the untreated G2 group (Fig. 4a). In addition, the superoxide level significantly decreased in the Mito-TEMPO-treated G2 group and its expression in the Mito-TEMPO-treated G2 group reduced as much as the G1 group (Fig. 4b). This was accompanied by a decrease in the formation of mitochondrial aggregation in the Mito-TEMPO-treated G2 group. Previous study showed that mitochondrial aggregation or abnormal mitochondrial morphologies impaired functions, released cytochrome c and leading to cell death^36^. Their report explained the relationship between changes of mitochondria morphology and mitochondrial functions. Based on their report, we speculated that reduction of mitochondria aggregation by Mito-TEMPO was involved in mitochondria functions on porcine embryos development. In addition, our result demonstrates that Mito-TEMPO enhanced embryo development rate in zygote G2 group through reduction of superoxide and mitochondrial aggregation.

**Figure 5:**
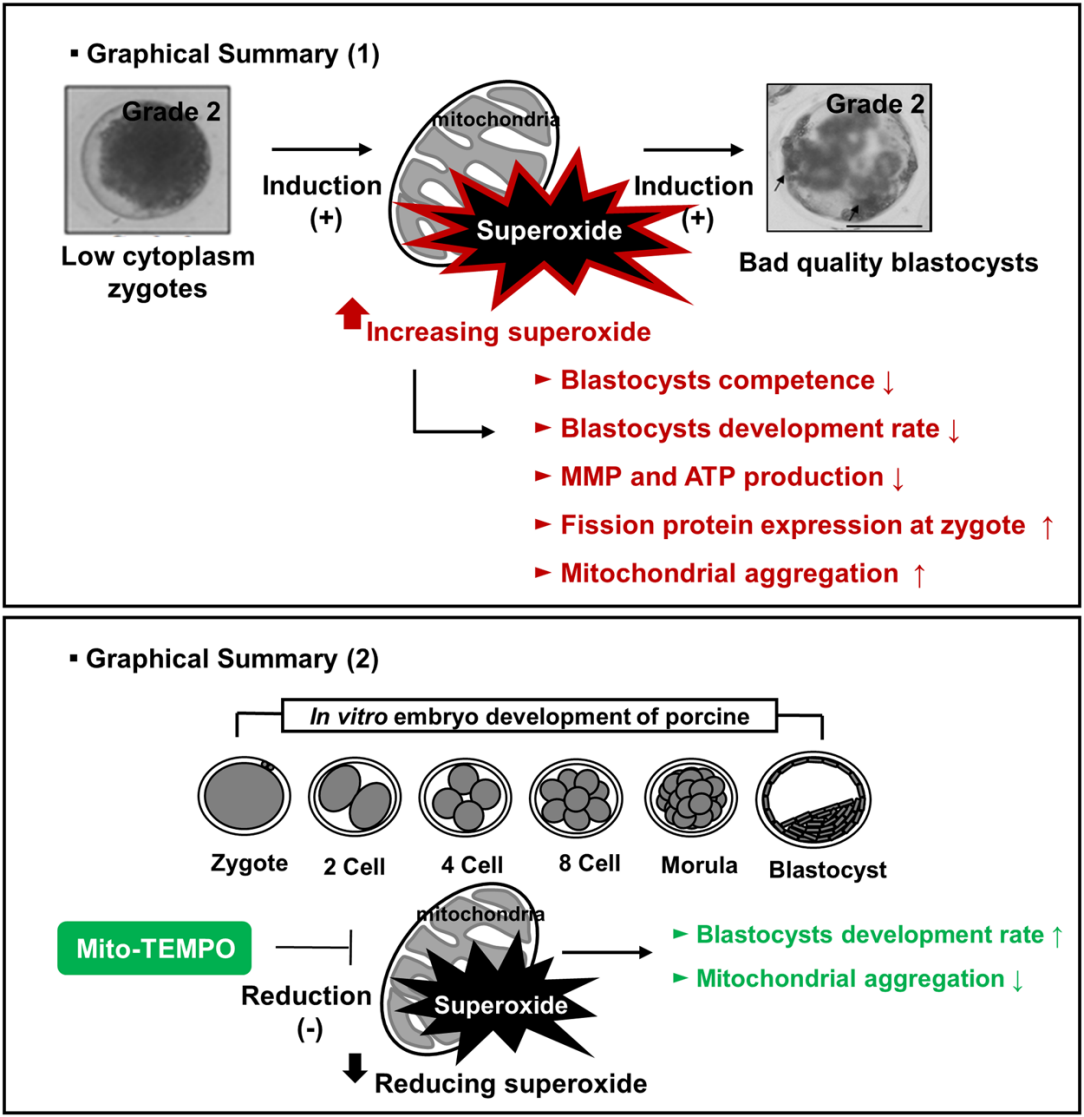
Schematic diagram illustrating the effect of Mito-TEMPO on porcine embryo development. Top panel; zygotes with low cytoplasm had increasing mitochondrial superoxide. In addition, high superoxide production in G2 embryos caused defective MMP and ATP production. Mitochondrial fission and aggregation increased in G2 embryos. Bottom panel; Zygotes of low cytoplasm cultured with Mito-TEMPO for early embryo development. Reduction of mitochondrial superoxide increases the blastocysts development rate and decreases mitochondrial aggregation in porcine.

In conclusion, our study provides the first evidence that Mito-TEMPO improves the developmental competence by reducing mitochondrial aggregation and superoxide in porcine embryos and blastocysts. As shown in Fig. 5 (top panel), increased superoxide induced low developmental competence, mitochondrial dysfunctions, and aggregation during porcine embryo development. In contrast, reduction of superoxide by Mito-TEMPO by acting as a mitochondrial superoxide scavenger improves blastocyst development and reduces mitochondrial aggregation (bottom panel). Therefore, controlling superoxide by Mito-TEMPO might be useful for future studies on the superoxide-mediated mechanism and mitochondrial redox signaling during preimplantation development of porcine embryos.

## Materials and Methods

### IVM, IVF and *in vitro* culture (IVC)

Unless noted otherwise, all chemicals used in the present study were purchased from Sigma-Aldrich Korea (St. Louis, MO, USA). We performed IVM as described by Yeon *et al*.^37^. Porcine ovaries were obtained from non-pregnant sows in local slaughterhouse and were transported to the laboratory in 0.9% saline supplemented with 75 μg/ml penicillin G sodium salt at 30–35 °C. Immature cumulus-oocyte complexes (COCs) were then aspirated from 3–6 mm follicles using a disposable syringe with an 18-gauge needle. Undamaged COCs with similar quality cytoplasm and surrounding cumulus cells were collected using mouth pipetting, then washed three times in tyrode’s lactate-N-2-hydroxyethylpiperazine-N’2-ethanesulfonic acid (TL-HEPES) medium. Next, 50 immature COCs were matured in 500 μl of IVM medium at 38.5 °C under 5% CO_2_. NCSU 23 medium supplemented with 10% follicular fluid, 0.57 mM cysteine, 10 ng/ml β-mercaptoethanol, 10 ng/ml epidermal growth factor (EGF), 10 IU/ml pregnant mare serum gonadotropin (PMSG) and 10 IU/ml human chorionic gonadotropin (hCG) were used for oocyte maturation. After culturing for 22 h, COCs were washed three times and then further cultured in PMSG and hCG-free maturation medium for 22 h. The IVF medium used modified tris-buffered medium (mTBM) containing 2.5 mM caffeine sodium benzoate and 1 mg/ml bovine serum albumin (BSA). Fresh semen was kindly supplied by AI company (Darby Porcine AI Center, Anseong, Korea) and kept at 17 °C for 5 days. Spermatozoa were washed three times by centrifugation in phosphate buffered saline (PBS) supplemented with 1 mg/ml BSA, 100 mg/ml penicillin G and 75 mg/ml streptomycin sulfate. After washing, spermatozoa were re-suspended in mTBM. Matured oocytes were denuded by softly pipetting in 0.1% hyaluronidase, then placed into 48 μl of mTBM under mineral oil. Next, 2 μl of diluted spermatozoa were added to a 48 μl drop of medium containing 15 oocytes to give a final concentration of 1.5 × 10^5^ sperm/ml. Finally, oocytes were co-incubated with the spermatozoa for 6 h at 38.5 °C under 5% CO_2_. Then, we divided zygotes into two groups (Grade 1: G1 and Grade 2: G2) according to the cytoplasm components at the zygote stage. When the total cytoplasmic area was 100%, we determined G1 (>90%) and G2 (<90%) groups according to cytoplasm. Then, embryos were cultured in 50 μl drops porcine zygote medium-3 (PZM-3) with 3 mg/ml BSA at 38.5 °C under 5% CO_2_. After culturing 48 hours, cleaved embryos were transferred to 50 μl drop of fresh PZM-3 medium and cultured for 4 days. Blastocyst formation was evaluated after 6 days of culture. Mito-TEMPO (0.1 μM) was added to the culture medium during the IVC periods.

### Oil Red O staining

Washed zygotes were fixed using 2.5% glutaraldehyde for 1 h at room temperature. The fixed zygotes were washed and incubated with 60% isopropanol. Then, Lipid drops were stained for 10 minutes at room temperature with filtered working solution (6:4; 350 mg/ml Oil Red O in isopropanol: diH_2_O). Stained zygotes were washed and then extracted Oil Red O dye in 100% isopropanol at room temperature. Extracted dye was measured with 500 nm using the Infinite M200pro microplate reader. (TECAN, Männedorf, Switzerland).

### TUNEL assay

Blastocysts were fixed in 2.5% glutaraldehyde in PBS for overnight at 4 °C. Terminal deoxynucleotidyl transferase-mediated dUTP nick end-labeling (TUNEL) analysis detected cellular apoptosis using an *in-situ* Cell Death Detection Kit (Roche Diagnostics GmbH, Mannheim, Germany) according to the manufacturer’s instructions. The fixed blastocysts were permeabilized by 0.5% Triton X-100 for 30 min at room temperature. Then blastocysts were incubated in TUNEL reaction solution for 1 h at 38.5 °C. Then stained blastocyst were washed and mounted on slides glass with DAPI solution. The stained blastocysts were examined by an A1 epifluorescence microscope (Zeiss, Jena, Germany).

### Analysis of MMP, Mitotracker, intracellular, and mitochondrial ROS

Embryos were washed three times with 0.1% polyvinyl alcohol (PVA) in PBS and incubated IVC medium with JC-1 (100:1) (Cyman Chemical, MI, USA) for 30 min at 38.5 °C. Then, washed embryos were fixed in 2.5% glutaraldehyde in PBS for overnight at 4 °C. The aggregated form (J-aggregate) of mitochondria emitted red fluorescence and the fluorescence of monomers form (J-monomer) indicated green fluorescence. Washed embryos were incubated in IVC medium with 0.8 μM Mitotracker green (Invitrogen, CA, USA), 0.4 μM MitoSOX red (Invitrogen, CA, USA) and 5 μM DCF-DA (Molecular Probes, Eugene, OR, USA) for 30 min at 38.5 °C. The fluorescence images of JC-1 and MitoSOX in the embryos were acquired using a LSM 700 confocal microscope (Zeiss, Jena, Germany). The intensity of DCF-DA was measured using an A1 epifluorescence microscope (Zeiss, Jena, Germany) and Mitotracker images were acquired by iRiS^TM^ Digital Cell Image System (Logos Biosystems, Gyeonggi-do, South Korea).

### Measurement of ATP

The concentration of ATP in embryos was determined by a bioluminescence assay using an ATP determination kit (Invitrogen, CA, USA). We made a standard solution according to the instructions of the manufacturer. Then 40 embryos were homogenized with 10 μl RIPA buffer and 90 μl standard buffer was added. The total reacted solution were transferred 200 μl per well into white 96-well plate and followed 10 min of incubation at room temperature. Luminescence intensity was measured using a luminometer. (InfiniteM200pro, Tecan, Männedorf, Switzerland).

### Western blot analysis

Cultured 40 embryos lysates in PRO-PREP protein lysis buffer (iNtRON, Daejeon, Korea) were prepared. Embryo lysates were separated by 10% SDS-PAGE. After electrophoresis, the separated proteins were transferred to pure nitrocellulose membranes (Pall Life sciences, NY, USA). After blocking, the membranes were incubated anti-phospho-Drp1-Ser616 (Cell signaling, MA, USA), Drp1 (Santa Cruz Biotechnology, CA, USA) and β-actin (Santa Cruz). The membranes were incubated with a secondary HRP-conjugated anti-rabbit/mouse IgG (Thermo, Scientific, MA, USA). Antibody binding was detected using the ECL kit (Advansta, CA, USA) according to the manufacturer’s instructions.

### Statistical analysis

All percentage data obtained in the present study were presented as the mean ± standard deviation (SD). Moreover, Western blot and image experiments made in triplicate and all values were presented as the mean standard error of the mean (SEM). All results were analyzed using either a one-way ANOVA followed Dunnett’s Multiple Comparison Test or *t*-tests. All data were performed using the GraphPad Prism 5.0 software package (San Diego, CA, USA). Histogram values of densitometry were measured by image J software (NIH, USA). Differences were considered significant at * p < 0.05; ** < 0.01; *** < 0.001.

### Data availability

All data generated or analysed during this study are included in this published article (and its Supplementary Information files).

## Electronic supplementary material

Supplementary Information
