# Supplementary material for: Mito-TEMPO improves development competence by reducing superoxide in preimplantation porcine embryos
Source: Sci Rep. 2018 Jul 4;8:10130. doi: 10.1038/s41598-018-28497-5 (PMC6031607; doi:10.1038/s41598-018-28497-5)
Supplement: Supplementary file 1 — Supplementary Information [file 41598_2018_28497_MOESM1_ESM.docx]

**Mito-TEMPO improves development competence by reducing superoxide in preimplantation porcine embryos**

**Seul-Gi Yang ^a,1^, Hyo-Jin Park^a,1^, Jin-Woo Kim^a^, Jae-Min Jung^a^, Min-Ji Kim^a^, Ho-Guen Jegal^a^, In-Su Kim^a^, Man-Jong Kang^b^, Gabbine Wee^c^, Hee-Young Yang^c^, Yun-Han Lee^d^, Ji-Hae Seo^e^, Sun-Uk Kim^f^, Deog-Bon Koo^a,*^**

^a^ Department of Biotechnology, College of Engineering, Daegu University, 201 Daegudae-ro, Jillyang, Gyeongsan, Gyeongbuk 38453, Republic of Korea

^b^ Department of Animal Science, College of Agriculture and Life Sciences, Chonnam National University, Gwangju 61186, Republic of Korea

^c^ Laboratory Animal Center, Daegu-Gyeongbuk Medical Innovation Foundation (DGMIF), 80 Cheombok-ro, Dong-gu, Daegu 41061, Republic of Korea

^d^ Department of Molecular Medicine, Keimyung University School of Medicine, Daegu 42601, Republic of Korea

^e^ Department of Biochemistry, Keimyung University School of Medicine, Daegu 42601, Republic of Korea

^f^ National Primate Research Center & Futuristic Animal Resource and Research Center, Korea Research Institute of Bioscience and Biotechnology, Ochang, Chungbuk 28116, Republic of Korea

^*^ Corresponding author at: Department of Biotechnology, College of Engineering, Daegu University, 201 Daegudae-ro, Jillyang, Gyeongsan, Gyeongbuk 38453, Republic of Korea

E-mail addresses: dbkoo@daegu.ac.kr (Deog-Bon Koo)

^1^ The first two authors contributed equally to this work


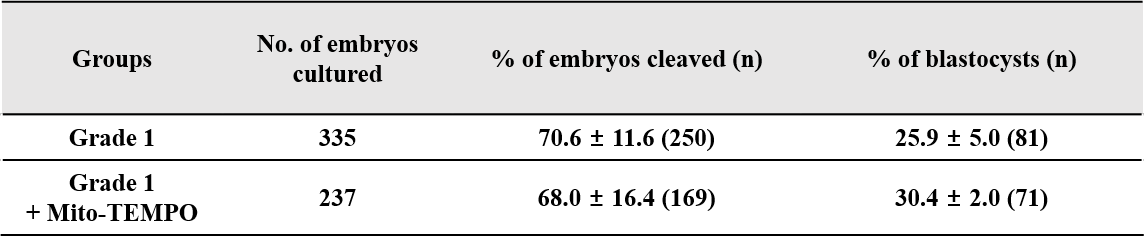

**Supplementary Figure S1.** Development rate by Mito-TEMPO treatment in G1 embryos. Data are expressed as means ± SD of three independent experiments.


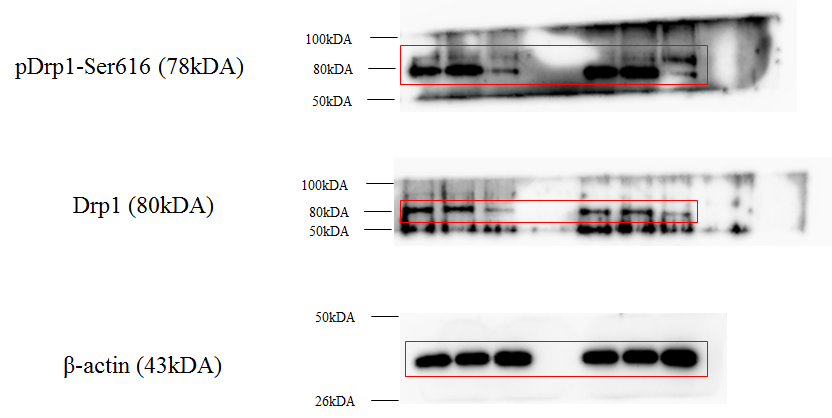


**Supplementary Figure S2.** The original figure for the bands of pDrp1-Ser616 (78 kDA), Drp1 (80 kDA), and β-actin (43kDA) for the Western blot experiments.

**Supplementary Table S1.** Effects of Mito-TEMPO on porcine oocytes maturation.

| TEMPO  (μM) | No. of oocytes examined | % of oocytes (n) | | | |
| --- | --- | --- | --- | --- | --- |
|  |  | GV | GVBD | M I | M II |
| Con | 251 | 2.9 ± 4.0 (7) | 1.1 ± 1.5 (4) | 16.5 ± 8.3 (42) | 79.6 ± 12.4 (198) ^a^ |
| 0.1 | 231 | 3.1 ± 3.1 (9) | 0.0 ± 0.0 (0) | 12.7 ± 7.9 (32) | 84.2 ± 10.9 (190) ^b^ |
| 0.5 | 247 | 0.9 ± 2.0 (5) | 1.9 ± 1.4 (4) | 16.6 ± 8.1 (43) | 80.6 ± 8.2 (195) ^a^ |
| 1.0 | 242 | 3.0 ± 2.0 (7) | 0.0 ± 0.0 (0) | 21.1 ± 15.2 (48) | 75.9 ± 15.4 (187) ^a^ |

Data are expressed as means ± SD of three independent experiments. Different superscript letters denote significant differences (p < 0.05).
